# Mitochondrial 8-hydroxy-2′-deoxyguanosine and coronary artery disease in patients with type 2 diabetes mellitus

**DOI:** 10.1186/s12933-020-00998-6

**Published:** 2020-02-19

**Authors:** Xue-bin Wang, Ning-hua Cui, Xia’nan Liu, Xin Liu

**Affiliations:** 1 Department of Clinical Laboratory, The First Affiliated Hospital of Zhengzhou University, Jianshe East Road No. 1, Zhengzhou, 450000 Henan China; 2 Zhengzhou Key Laboratory of Children’s Infection and Immunity, Children’s Hospital Affiliated to Zhengzhou University, Zhengzhou, 450000 Henan China

**Keywords:** Mitochondrial 8-OHdG, Coronary artery disease, Type 2 diabetes mellitus, Clinical outcomes after revascularization

## Abstract

**Background:**

Little is known about whether mitochondria 8-hydroxy-2′-deoxyguanosine (8-OHdG), a biomarker of mitochondrial DNA (mtDNA) oxidative damage, contributes to the development of coronary artery disease (CAD) in diabetic patients. Here, we explored the associations of mtDNA 8-OHdG in leukocytes with obstructive CAD, coronary stenosis severity, cardiovascular biomarkers, and 1-year adverse outcomes after coronary revascularization in patients with type 2 diabetes mellitus (T2DM).

**Methods:**

In a total of 1920 consecutive patients with T2DM who underwent coronary angiography due to symptoms of angina or angina equivalents, the presence of obstructive CAD, the number of diseased vessels with ≥ 50% stenosis, and modified Gensini score were cross-sectionally evaluated; the level of mtDNA 8-OHdG was quantified by quantitative PCR. Then, 701 of 1920 diabetic patients who further received coronary revascularization completed 1-year prospective follow-up to document major adverse cardiovascular and cerebral events (MACCEs). In vitro experiments were also performed to observe the effects of mtDNA oxidative damage in high glucose-cultured human umbilical vein endothelial cells (HUVECs).

**Results:**

Cross-sectionally, greater mtDNA 8-OHdG was associated with increased odds of obstructive CAD (odds ratio [OR] 1.38, 95% CI confidence interval 1.24–1.52), higher degree of coronary stenosis (number of diseased vessels: OR 1.29, 95% CI 1.19–1.41; modified Gensini scores: OR 1.28, 95% CI 1.18–1.39), and higher levels of C-reactive protein (β 0.18, 95% CI 0.06–0.31) after adjusting for confounders. Sensitivity analyses using propensity score matching yielded similar results. Stratification by smoking status showed that the association between mtDNA 8-OHdG and obstructive CAD was most evident in current smokers (*P*_interation_ < 0.01). Prospectively, the adjusted hazards ratio per 1-SD increase in mtDNA 8-OHdG was 1.59 (95% CI 1.33–1.90) for predicting 1-year MACCEs after revascularization. In HUVECs, exposure to antimycin A, an inducer for mtDNA oxidative damage, led to adverse alterations in markers of mitochondrial and endothelia function.

**Conclusion:**

Greater mtDNA 8-OHdG in leukocytes may serve as an independent risk factor for CAD in patients with T2DM.

## Introduction

Patients with type 2 diabetes mellitus (T2DM) exhibit a high prevalence of coronary artery disease (CAD) and cardiac deaths [1, 2]. T2DM-related variables, including long diabetes duration [3], high glycemic variability [4, 5], and elevated glycated hemoglobin (HbA1c) [6], are also considered as potent predictors for cardiovascular events in large-scale prospective studies. The pathogenesis of clinically overt CAD in T2DM, while not fully elucidated, has been related to the excess production of reactive oxygen species (ROS), which may active proinflammatory pathways, reduce nitric oxide bioavailability, and consequently induce diabetic endothelial dysfunction [7, 8]. Mitochondria, as central regulators of energy metabolism, are primary cellular sources and targets of oxidants associated with vascular disease in T2DM [9].

Mitochondrial DNA (mtDNA) is more vulnerable to the accumulation of ROS-mediated damage than nuclear DNA, in part because of its close proximity to the mitochondrial oxidative phosphorylation chain, and because it lacks protective histones and efficient DNA repair mechanisms [10]. Under diabetic conditions, oxidative damage to the mtDNA may be an important consequence of excess ROS production [11], inducing a loss of mitochondrial networks, decreased expression of mitochondrial respiratory complexes, and consequent T2DM-related atherosclerosis [12–14]. Among numerous types of mtDNA oxidative lesions, the formation of 8-hydroxy-2′-deoxyguanosine (8-OHdG) is the most abundant one, reflecting a steady-state level of oxidative damage occurring within mtDNA [15]. In kidney of diabetic rats, levels of 8-OHdG were up-regulated in mtDNA but not in nuclear DNA [16]. In population studies, we and others previously showed that leukocyte mtDNA copy number variation (mtDNA-CN), as an indirect measure of mtDNA damage [17], inversely correlated with CAD risk and severity independent of diabetic status [18, 19]. However, little is known about whether 8-OHdG in mtDNA, a more direct marker of oxidative DNA damage, contributes to the development of CAD in patients with T2DM.

Hence, in this cross-sectional study, we enrolled a total of 1920 consecutive patients with T2DM to assess the associations of mitochondrial 8-OHdG in leukocytes with the presence of obstructive CAD, the severity of coronary artery stenosis, and cardiometabolic phenotypes. Then, 701 of 1920 patients who received percutaneous or surgical revascularization were prospectively followed for 1 year to investigate the prognostic value of mitochondrial 8-OHdG on clinical outcomes after revascularization. Finally, we performed in vitro experiments to assess the effect of antimycin A (AMA)-induced mtDNA oxidative damage on markers of mitochondrial and endothelial dysfunction in high glucose-cultured human umbilical vein endothelial cells (HUVECs).

## Methods

### Study population

**Fig. 1:**
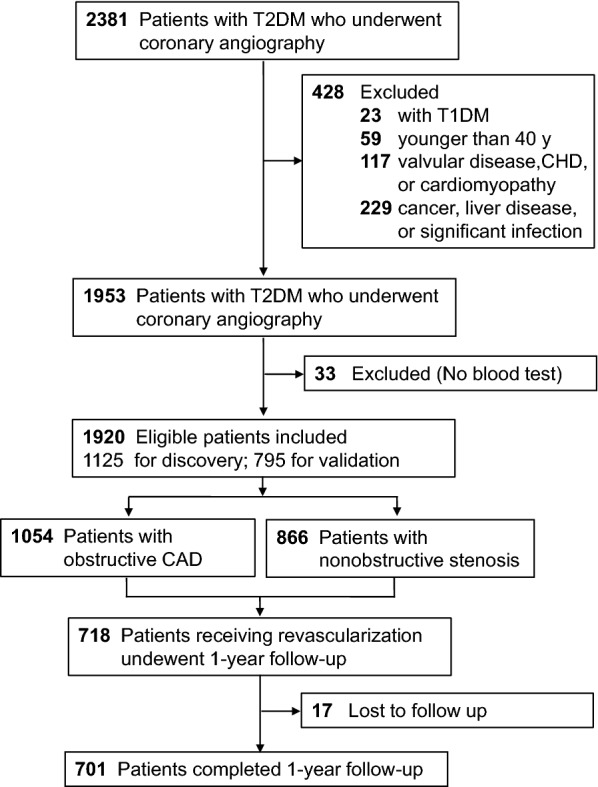
Flow chart of our study

From May 2016 to May 2018, 2381 consecutive patients with T2DM who underwent coronary angiography due to symptoms of angina or angina equivalents were cross-sectionally evaluated in a discovery cohort from The First Affiliated Hospital of Zhengzhou University (at Henan province, northern China) and in a validation cohort from Zhongnan Hospital of Wuhan University (at Hubei province, central China). Patients were excluded if they (1) were younger than 40 years (n = 59); (2) did not complete blood tests (n = 33); (3) had a history of type 1 diabetes (n = 23); (4) had a history of systemic disease including cancer of any type, liver disease, and clinically significant infection (n = 229); (5) had a history of cardiac disease including valvular disease, congenital heart disease, and cardiomyopathy (n = 117). After excluding 461 cases due to exclusion criteria, a total of 1920 patients with T2DM were finally included, involving 1125 cases in a discovery cohort and 795 cases in a validation cohort (Fig. 1). The diagnosis of T2DM was based on the American Diabetes Association 2014 criteria: fasting plasma glucose (FPG) ≥ 7.0 mmol/L and/or HbA1c ≥ 6.5% and/or 2-h post-challenge plasma glucose ≥ 11.1 mmol/L and/or ongoing therapy for T2DM [20]. Obstructive CAD was defined as the maximum luminal stenosis of ≥ 50% in any segment of major coronary arteries [21, 22].

The severity of coronary artery stenosis was quantified by the number of diseased vessels with ≥ 50% stenosis and modified Gensini scores. We first categorized each patient by the number of diseased vessels with ≥ 50% stenosis in a single, double, or triple-vessel distribution. We defined vessel distribution by the left anterior descending artery and its branches, the left circumflex artery and its branches, and the right coronary artery and its branches. Patients with ≥ 50% stenosis of left main coronary artery were classified into the 3-vessel obstructive CAD group [22]. So we finally created four categories of CAD extent: non-obstructive CAD, 1-, 2-, 3-vessel/left main obstructive CAD. In the Gensini scoring system [23], angiographic stenosis of each coronary segment was first scored according to the degree of luminal narrowing: 1 for 0–25%, 2 for 26–50%, 4 for 51–75%, 8 for 76–90%, 16 for 91–99%, and 32 for 100% stenosis. Then a multiplier was assigned to each segment depending on the prognostic importance of the area supplied by that segment: 5 for left main coronary artery, 2.5 for proximal left anterior descending coronary artery (LAD) and proximal left circumflex branch, 1.5 for mid-segment of LAD, 0.5 for second diagonal branch and posterolateral branch, and 1 for other branches. Finally, the weighted score of each lesion was summed to give final Gensini scores.

During the study period, 718 of 1920 patients with T2DM underwent percutaneous coronary intervention (PCI) or coronary artery bypass grafting (CABG) due to the presence of indications for revascularization [24, 25]. These 718 patients were followed up for 12 months after revascularization to track major adverse cardiovascular and cerebrovascular events (MACCEs) as the primary outcome. MACCE was defined as a composite of all-cause death, repeat revascularization, non-fatal myocardial infarction (MI), and non-fatal stroke (Additional file 1: Methods) [26]. The second outcome was any cause of cardiac death. All outcomes of interest were ascertained through telephone interviews with participants and verification by checking medical records and administrative billing codes for readmissions at 30 days and 6, 9, 12 months after revascularization. In total, 701 patients (97.6%) were completely followed for clinical outcomes up to 12 months or deaths. The study protocol followed the Declaration of Helsinki and was approved by the Ethics Committees of participating centers. All participants signed written informed consent.

### Collection and definition of clinical variables

Detailed description of clinical variables has been presented in our previous studies [18, 27]. Briefly, demographic factors, including age, sex, smoking status (never, former, and current), alcohol drinking status (ever and never), diabetes duration, and type of diabetes management (lifestyle modification, oral agents, insulin, and oral agents plus insulin), were documented by checking participants’ medical records and questionnaires. Weight and height were measured on the day of enrollments, and body mass index (BMI) was coded into three categories: normal BMI (18–24.9 kg/m^2^), overweight (25–30 kg/m^2^), and obese (> 30 kg/m^2^). Dyslipidemia and hypertension were defined based on the previously published guidelines [28, 29].

Fasting blood samples were drawn on the day of angiography. FPG, low-density lipoprotein cholesterol (LDL-c), high-density lipoprotein cholesterol (HDL-c), total cholesterol (TC), triglycerides (TG), and C-reactive protein (CRP) were measured on a Cobas 8000 Analyzer (Roche Diagnostics, Germany) using standard methods. HbA1c was assayed by high-performance liquid chromatography on a Bio-Rad Hemoglobin Testing System. Plasma D-dimer was detected with latex enhanced immunoassay on an ACL TOP 300 Analyzer (Beckman, USA).

### Quantitative of the levels of 8-OHdG in leukocyte mtDNA

Leukocyte DNA was extracted using the QIAamp DNA minikit (Qiagen, MD, USA). The levels of mtDNA 8-OHdG were measured by quantitative PCR (qPCR) to amplify a fragment of mtDNA with or without treatment of human 8-oxoguanine DNA glycosylase 1 (hOGG1), as described by Lin et al. [30, 31]. Briefly, this method is based on the theory that pretreatment of hOGG1 would remove the 8-OHdG residues to form abasic sites that reduce the amplification efficacy [32]. So, the content of 8-OHdG in mtDNA is relative to the differences in amplification efficiency between hOGG1-treated and untreated DNA when the cycling number was in the linear amplification stage [30]. First, 100 ng of DNA sample was treated with or without 1 U of hOGG1 (TREVIGEN, MD, USA) in a mix containing 1.5 μL of 10× buffer NE, 8.5 μL of RNase free water, and 100 μg/L of bovine serum albumin, followed by an incubation of 1 h for 37 °C. Then, the treated and untreated samples were amplified by qPCR using a specific primer pair designed for the mitochondrial *ND1* gene in a reaction mixture containing ~ 25 ng of DNA sample, 2 μL of ddH_2_O, and 10 pM of each primer (F: 5′-CAC CCA AGA ACA GGG TTT GT-3′; R: 5′-TGG CCA TGG GAT TGT TGT TAA-3′). Each sample was assayed in duplicate, and the mean value of the ∆ct (treated ct − untreated ct) was calculated as the level of mtDNA 8-OHdG.

For quality control, four samples were included into each plate to analyze variability. The average CV across plates was 4.58%. For validation of qPCR data, we also measured the levels of 8-OHdG in mtDNA using a previously described ELISA assay based on mitochondria isolation, mtDNA extraction and digestion, and immunosorption [16] in 200 participants randomly selected from the whole cohorts. The bivariate correlation coefficient between qPCR and ELISA data was greater than 0.72 (Additional file 2: Fig. S1), suggesting that the levels of 8-OHdG determined by qPCR were reliable.

### In vitro experiment in HUVECs

HUVECs (ATCC Cat# PCS-100-010) were cultured in complete endothelial cell growth medium (EGM-2 Bulletkit, LONZA) containing high levels of glucose (25 mM d-glucose) in a humidified incubator with 5% CO_2_ at 37 °C. The cells were incubated for 72–96 h until fully confluent, and treated with or without 50 mM of AMA (Sigma, MO, USA), an inducer of mtDNA oxidative damage by specifically inhibiting mitochondrial complex 3 [33]. After 24 h of incubation, cells with or without exposure to AMA were separately collected.

For the measurement of mitochondrial membrane potential (∆ψm), cells were washed twice with PBS, and incubated with 200 μM of JC-1 (Invitrogen, CA, USA) for 20 min, as described by Widlansky et al. [34]. JC-1 is a cationic dye whose mitochondrial uptake is directly correlated with the magnitude of ∆ψm. Mitochondrial membrane potential was expressed as the JC-1 fluorescence intensities detected by the Microplate Reader at Ex/Em(green)/Em(red) of 485/538/590 nm. MtDNA 8-OHdG, mtDNA-CN, and mRNA expression of peroxisome proliferator-activated receptor coactivator 1a (*PGC*-*1α*, a master gene of mitochondrial biogenesis [35]) gene were determined using qPCR, as previously described [18, 36, 37]. Culture supernatants of HUVECs were collected to measure the levels of nitric oxide (NO), soluble vascular adhesion molecule 1 (sVCAM-1) and E-selectin using colorimetric kits (for NO) or ELISA (Invitrogen). We also performed a MTT assay (Sigma, MO, USA) to examine cell viability of HUVECs. Optical densities were measured at 562 nm with the results being normalized to untreated cells [33]. All in vitro experiments were performed in triplicate.

### Statistical analysis

We applied a *Z* transformation to standardize the distribution of mtDNA 8-OHdG across cohorts. The association of 8-OHdG in mtDNA with obstructive CAD was analyzed by logistic regression models with three progressive degrees of adjustment. Model 1 was a crude model without any confounders; model 2 was adjusted for age, sex, and cardiovascular risk factors including smoking habit, alcohol drinking habit, overweight/obesity, hypertension, and dyslipidemia; model 3 was additionally adjusted for T2DM-related variables including FPG, HbA1c, diabetes duration, and type of diabetes management. For each model, 8-OHdG in mtDNA was first analyzed as a continuous variable, and then as an ordinal variable based on its quartile distribution. We used the restricted cubic spline (RCS) to analyze the shape of the association between mtDNA 8-OHdG and CAD presence, with four equally spaced knots set at 5th, 35th, 65th, and 95th percentiles as recommended by Harrell et al. [38]. Sensitivity analyses were based on two statistical approaches: (1) stratification analysis assessing the effect modification by clinical covariates, (2) propensity score (PS) matching [39], which matched the distribution of covariates after using a 1:1 nearest neighbor matching (caliper = 0.05) to yield 673 pairs of participants.

We calculated the increases in Harrell’s C-statistic (i.e. ΔC-statistic) to assess the incremental value of adding mtDNA 8-OHdG separately to the 2013 ACC/AHA Pooled Cohorts Equations (PCE) [40] and the Framingham Risk Score (FRS) [41] in discriminating obstructive CAD. We computed the net reclassification improvement (NRI) as an alternative approach to evaluate risk classification ability when the risk estimates of obstructive CAD was categorized as low (< 20%), intermediate (20–50%), and high risk (> 50%). Finally, we calculated the integrated discrimination improvement (IDI), which integrates the continuous NRI over all possible cutoffs of risk estimates and mathematically corresponds to the difference in discrimination slopes of the two models in comparison [42]. The correlations of mtDNA 8-OHdG with coronary stenosis severity and cardiometabolic phenotypes were assessed by ordinal logistic regression and multivariable linear regression, respectively.

When assessing the prognostic value of mtDNA 8-OHdG on 1-year clinical outcomes after revascularization, we first used the time-dependent receiver operator characteristics (ROC) curve to screen the optimal cutoff value of mtDNA 8-OHdG with the highest combined sensitivity and specificity to predict MACCEs. Then, the Cox regression was performed to estimate hazard ratios (HRs) for the association between mtDNA 8-OHdG (as a continuous variable or as a dichotomous variable categorized by the ROC-derived cutoff value) and the incidence of MACCEs after adjusting for all confounders in model 3. For in vitro experiments, the Student t test was used to assess the differences in tested markers between AMA-treated and untreated HUVECs. All above analyses were conducted using STATA 12.0 (StataCorp, TC, USA). A statistical power was estimated using PS software 3.0 (Vanderbilt, TN, USA). A two-sided P < 0.05 was considered statistically significant.

## Results

### Characteristics of participants

Table 1 presents the clinical characteristics of all 1920 participants. Overall, type 2 diabetic patients with CAD were older, more likely to be smokers, obese, hypertensive, and dyslipidemic, and had higher levels of FPG, HbA1c, TC, LDL-c, and CRP than those without. After PS matching, a sub-cohort of 673 pairs of participants was screened. Within this cohort, all clinical characteristics were similar between CAD and non-CAD groups.

**Table 1:** Clinical characteristics of 1920 diabetic patients

Variables	All participants			After PS matching		
	Obstructive CAD (N = 1054)	Non-CAD (N = 866)	*P*^a^	Obstructive CAD (N = 673)	Non-CAD (N = 673)	*P*^a^
Age, years	62.8 ± 9.6	61.1 ± 10.7	< 0.001	62.3 ± 9.5	62.3 ± 10.8	0.977
Male, n (%)	583 (55.3)	480 (55.4)	0.960	360 (53.5)	373 (55.4)	0.477
BMI, mean (SD), kg/m^2^	25.2 ± 4.0	24.3 ± 2.6	< 0.001	24.7 ± 3.9	24.5 ± 2.6	0.204
BMI categories						
Normal	540 (51.2)	494 (57.0)	< 0.001	412 (61.2)	396 (58.8)	0.613
Overweight	372 (35.3)	317 (36.6)		116 (17.2)	118 (17.6)	
Obese	142 (13.5)	55 (6.4)		145 (21.6)	159 (23.6)	
Smoking, n (%)						
Never smoker	582 (55.2)	555 (64.1)	< 0.001	382 (56.8)	358 (53.2)	0.157
Former smoker	168 (15.9)	130 (15.0)		231 (34.3)	264 (39.2)	
Current smoker	304 (28.9)	181 (20.9)		60 (8.9)	51 (7.6)	
Alcohol drinkers, n (%)	343 (32.5)	248 (28.6)	0.065	210 (31.2)	196 (29.1)	0.406
FPG, mmol/L	7.3 ± 2.0	6.7 ± 1.8	< 0.001	7.0 ± 1.6	6.9 ± 2.0	0.386
HbA1c, (%)	8.1 ± 1.8	7.9 ± 1.7	0.031	8.0 ± 1.8	8.0 ± 1.7	0.779
Diabetes duration, years	10.3 ± 5.4	9.9 ± 5.7	0.110	10.1 ± 5.4	9.7 ± 5.7	0.105
Diabetes management						
Lifestyle modification	291 (27.6)	255 (29.4)	0.566	200 (29.7)	199 (29.6)	0.500
Oral agents only	332 (31.5)	283 (32.7)		206 (30.6)	229 (34.0)	
Insulin only	275 (26.1)	214 (24.7)		166 (24.7)	157 (23.3)	
Oral agents and insulin	156 (14.8)	114 (13.2)		101 (15.0)	88 (13.1)	
Dyslipidemia, n (%)	334 (31.7)	166 (19.2)	< 0.001	164 (24.4)	158 (23.5)	0.701
Cholesterol, mmol/L						
TC, mmol/L	4.95 ± 0.91	4.80 ± 0.85	< 0.001	4.88 ± 0.90	4.85 ± 0.87	0.194
LDL-c, mmol/L	3.28 ± 0.83	3.08 ± 0.68	< 0.001	3.16 ± 0.80	3.10 ± 0.67	0.123
HDL-c, mmol/L	1.12 ± 0.15	1.16 ± 0.21	0.318	1.09 ± 0.15	1.12 ± 0.21	0.756
TG, mmol/L	1.48 ± 0.85	1.36 ± 0.76	0.104	1.49 ± 0.84	1.43 ± 0.80	0.114
Hypertension, n (%)	619 (58.7)	338 (39.0)	< 0.001	330 (49.0)	318 (47.3)	0.513
Blood pressure						
SBP, mmHG	138.2 ± 21.4	134.8 ± 19.5	< 0.001	136.7 ± 25.0	135.4 ± 19.1	0.157
DBP, mmHG	88.0 ± 18.4	85.1 ± 12.0	< 0.001	86.4 ± 18.1	85.9 ± 11.4	0.109
CRP, mg/L	1.57 ± 1.01	1.10 ± 0.86	< 0.001	1.34 ± 0.94	1.30 ± 0.87	0.400
D-dimer, mg/L	0.98 ± 0.93	0.91 ± 0.82	0.077	0.93 ± 0.95	0.91 ± 0.79	0.228

Data are presented as the mean ± SD or n (%) as appropriate

*BMI* body mass index, *FPG* fasting plasma glucose, *HbA1c* glycosylated haemoglobin, *TC* total cholesterol, *LDL*-*c* low-density lipoprotein cholesterol, *HDL*-*c* high-density lipoprotein cholesterol, *TG* triglycerides, *SBP* systolic blood pressure, *DBP* diastolic blood pressure, *CRP* C-reactive protein, *CAD* coronary artery disease, *PS* propensity score

^a^P values were obtained from the student t test for continuous variables, and the Pearson χ2 test for categorical variables

### mtDNA 8-OHdG and obstructive CAD in diabetic patients

**Fig. 2:**
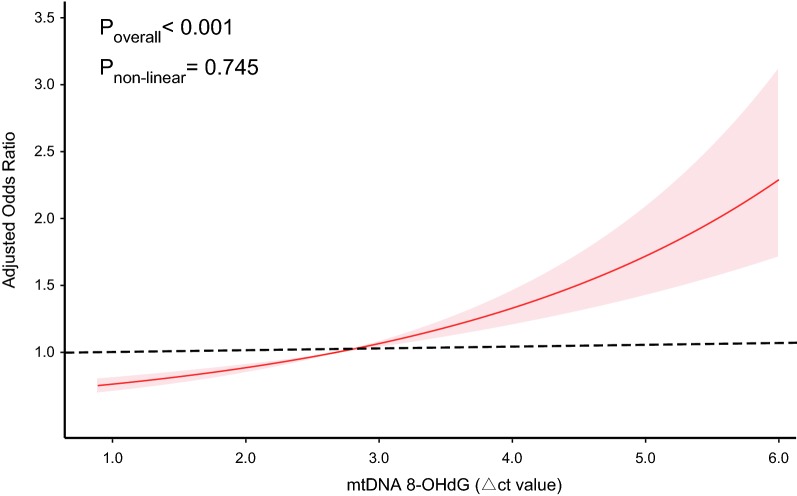
Restricted cubic spline analysis for assessing the shape of the association between mtDNA 8-OHdG and CAD presence in patients with T2DM after adjusting for age, sex, smoking habit, alcohol drinking habit, overweight/obesity, hypertension, dyslipidemia., FPG, HbA1c, diabetes duration, and type of diabetes management

In the whole cohort of 1920 diabetic patients, each 1-SD increase (0.87 ∆ct) in mtDNA 8-OHdG was associated with a 1.41-fold (95 confidence interval [95% CI] 1.28–1.56, Table 2) increased odds of CAD in the crude model. When further adjusted for age, sex, and, cardiovascular risk factors (model 2), the odds ratio (OR) for CAD was attenuated to 1.38 (95% CI 1.25–1.53) per 1-SD increase in mtDNA 8-OHdG. Additional adjustment for FPG, HbA1c, diabetes duration, and type of diabetes management (model 3) did not modify the magnitude of the association (OR = 1.38). Assuming an OR of 1.38 and a type I error of 0.05, the whole population could provide a statistical power of 98.3% to address the association. When analyzed with the bottom quartile of mtDNA 8-OHdG as the reference, the OR for CAD was 0.93 in the second quartile, 1.32 in the third quartile, and 2.41 in the top quartile, with a significant dose–response trend observed (*P*_trend_ < 0.001, Table 2). Similarly, the RCS analysis suggested that mtDNA 8-OHdG yielded much greater and linear-shaped increases in CAD rates across its ranges (*P*_non-linearity_ = 0.745, Fig. 2).

**Table 2:** Association of mtDNA 8-OHdG with CAD risk in patients with T2DM

mtDNA 8-OHdG	Obstructive CAD N (%)	Non-CAD N (%)	OR (95%CI)		
			Model 1^a^	Model 2^b^	Model 3^c^
All participants					
Each 1-SD increase	1054	866	1.41 (1.28–1.56)	1.38 (1.25–1.53)	1.38 (1.24–1.52)
Quartiles					
Q1 (< 2.84)	232 (22.0)	261 (30.1)	1 (Reference)	1 (Reference)	1 (Reference)
Q2 (2.84–3.20)	224 (21.3)	251 (29.0)	1.00 (0.78–1.29)	0.94 (0.72–1.22)	0.93 (0.71–1.22)
Q3 (3.20–3.53)	267 (25.3)	213 (24.6)	1.41 (1.10–1.82)	1.33 (1.02–1.73)	1.32 (1.01–1.72)
Q4 (> 3.53)	331 (31.4)	141 (16.3)	2.64 (2.03–3.44)	2.42 (1.84–3.19)	2.41 (1.82–3.19)
*P* for trend			< 0.001	< 0.001	< 0.001
After PS matching					
Each 1-SD increase	673	673	1.39 (1.24–1.56)	1.39 (1.24–1.55)	1.39 (1.24–1.55)
Quartiles					
Q1 (< 2.84)	149 (22.1)	187 (27.8)	1 (Reference)	1 (Reference)	1 (Reference)
Q2 (2.84–3.17)	148 (22.0)	190 (28.2)	0.98 (0.72–1.33)	0.97 (0.71–1.31)	0.94 (0.69–1.28)
Q3 (3.17–3.52)	163 (24.2)	176 (26.2)	1.16 (0.86–1.57)	1.16 (0.86–1.57)	1.17 (0.86–1.58)
Q4 (> 3.52)	213 (31.7)	120 (17.8)	2.22 (1.63–3.04)	2.19 (1.60–3.00)	2.18 (1.60–2.99)
*P* for trend			< 0.001	< 0.001	< 0.001

*PS* propensity score, *8*-*OHdG* 8-hydroxy-2′-deoxyguanosine, *CAD* coronary artery disease

^a^Crude model without any confounders

^b^Model 2 was adjusted for age, sex, smoking habit, alcohol drinking habit, overweight/obesity, hypertension, and dyslipidemia

^c^Model 3 was adjusted for age, sex, smoking habit, alcohol drinking habit, overweight/obesity, hypertension, dyslipidemia., FPG, HbA1c, diabetes duration, and type of diabetes management

**Fig. 3:**
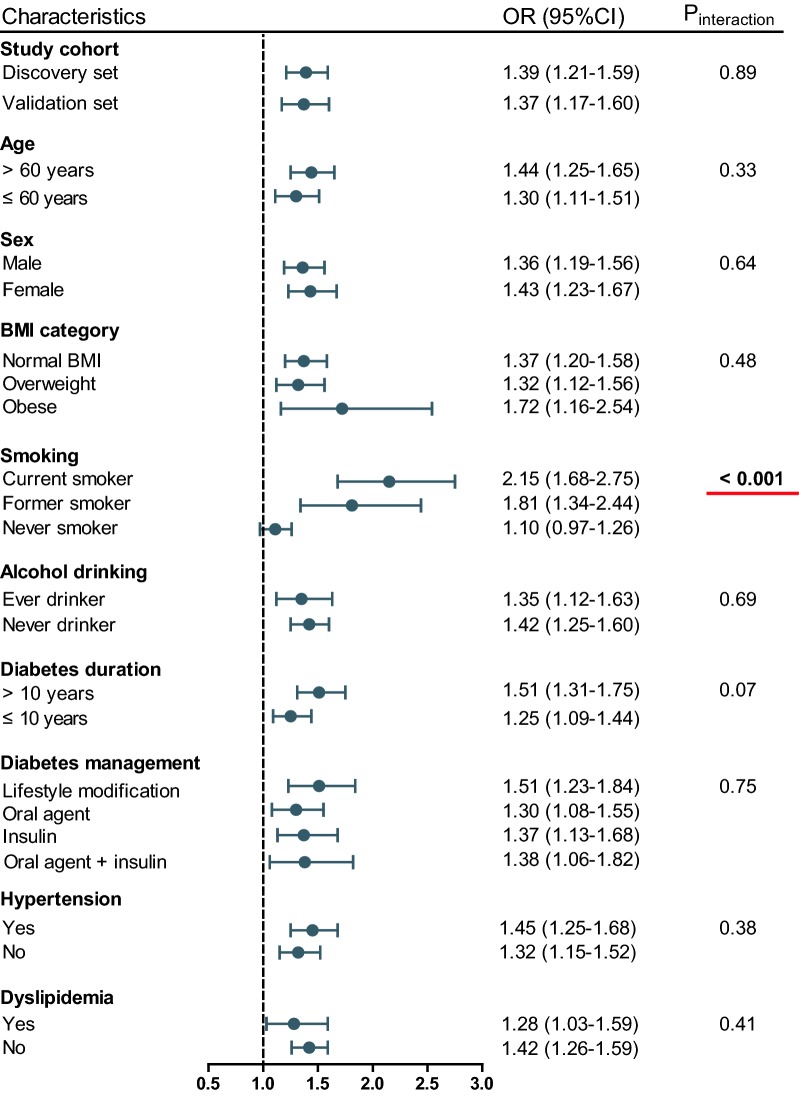
Association between continuous mtDNA 8-OHdG and CAD presence stratified by different clinical covariates in patients with T2DM. *P*_interaction_ was obtained from the χ2-based Q test

When stratified by smoking status, the OR for CAD per 1-SD increase of mtDNA 8-OHdG gradually increased from 1.10 in never smokers, to 1.81 in former smokers, and ultimately to 2.15 in current smokers *(P*_interaction_ < 0.001, Fig. 3). When stratified by study cohorts and other clinical covariates, no effect modification was observed. In the PS-matched cohort of 673 pairs of participants with balanced distributions of clinical covariates, mtDNA 8OHdG, either as a continuous variable or as an ordinal variable, continued to be positively associated with an increased odds of CAD, to a similar degree as in the whole cohort (Table 2).

### mtDNA 8-OHdG and risk discrimination and reclassification for CAD in diabetic patients

Adding mtDNA 8-OHdG to the 2013 ACC/AHA PCE model significantly improved risk discrimination and reclassification for CAD events, measured by ΔC-statistic (0.024), continuous NRI (0.299) and IDI (0.020, all *P* < 0.001). When categorizing the overall NRI with cutoffs at 20% and 50%, the addition of mtDNA 8-OHdG properly upclassified 45 individuals with CAD events, and appropriately downclassified 52 individuals without CAD (categorical NRI = 0.103, *P* < 0.001, Table 3). Similarly, when mtDNA 8-OHdG was added to the FRS, there were significant improvements in discrimination (ΔC-statistic: 0.010, *P* = 0.003) and risk reclassification (categorical NRI = 0.059, continuous NRI = 0.196, IDI = 0.014, all *P* < 0.001) for CAD events (Table 3). All models including mtDNA 8-OHdG were well-calibrated, with *P* values of the Hosmer–Lemeshow test of > 0.1.

**Table 3:** Discrimination and reclassification improvements of mtDNA 8-OHdG beyond the 2013 ACC/AHA PCE and the FRS in 1920 patients with T2DM

Risk estimates	ACC/AHA 2013 PCE + mtDNA 8-OHdG					FRS + mtDNA 8-OHdG				
	< 20%	20–50%	> 50%	Total	Reclassified	< 20%	20–50%	> 50%	Total	Reclassified
Individuals without obstructive CAD, No.										
< 20%	268	23	0	291	8%	352	10	0	362	3%
20–50%	68	275	41	384	28%	59	191	14	264	28%
> 50%	0	48	143	191	25%	0	27	213	240	11%
Total	336	346	184	866		411	228	227	866	
Individuals with obstructive CAD, No.										
< 20%	125	30	3	158	21%	105	7	0	112	6%
20–50%	35	288	82	405	29%	30	205	41	276	26%
> 50%	0	35	456	491	7%	0	31	635	666	5%
Total	160	353	541	1054		135	243	676	1054	

	Estimate (95% CI)	*P* value	Estimate (95% CI)	*P* value
Δ C-statistic	0.024 (0.013–0.035)	< 0.001	0.010 (0.003–0.017)	0.003
IDI	0.020 (0.014–0.026)	< 0.001	0.014 (0.001–0.019)	< 0.001
NRI continuous	0.299 (0.210–0.388)	< 0.001	0.196 (0.107–0.286)	< 0.001
NRI categorical	0.103 (0.064–0.142)	< 0.001	0.059 (0.029–0.090)	< 0.001

*PCE* ACC/AHA Pooled Cohorts Equations, *FRS* the Framingham Risk Score, *IDI* integrated discrimination improvement, *NRI* integrated discrimination improvement

### mtDNA 8-OHdG and coronary stenosis severity in diabetic patients

For each diabetic patient, the severity of coronary stenosis was ordinally scored as the number of diseased vessels with stenosis of ≥ 50% and the quartiles of modified Gensini scores. Ordinal logistic regression showed that each 1-SD increase in mtDNA 8-OHdG was associated with a 29% higher odds of having more diseased vessels and a 28% greater odds of being a higher quartile of Gensini scores after model 3 adjustment (both *P* < 0.001, Table 4). When analyzing mtDNA 8-OHdG as an ordinal variable, the adjusted OR in the top versus bottom quartile of mtDNA 8-OHdG was 1.91 (95% CI 1.50–2.42) for having more diseased vessels, and 1.94 (95% CI 1.54–2.44) for being higher quartiles of Gensini scores. Sensitivity analyses based on PS matching continuously observed the significant associations of mtDNA 8-OHdH with the number of diseased vessels and Gensini scores (Table 4).

**Table 4:** Associations of mtDNA 8-OHdG with coronary stenosis severity as determined by the number of disease vessels and quartiles of modified Gensini scores

mtDNA 8-OHdG	No. of diseased vessels with > 50% stenosis				OR (95% CI)^a^	Quartiles of modified Gensini scores^b^				OR (95% CI)^a^
	Non-CAD	1	2	3/LMCAD		Q1	Q2	Q3	Q4	
All participants										
Each 1-SD increase	866	448	337	269	1.29 (1.19–1.41)	486	478	482	474	1.28 (1.18–1.39)
Quartiles, N (%)										
Q1	261 (30.1)	97 (21.7)	86 (25.5)	49 (18.2)	1 (Reference)	149 (30.6)	137 (28.7)	117 (24.3)	90 (19.0)	1 (Reference)
Q2	251 (29.0)	108 (24.1)	59 (17.5)	57 (21.2)	0.92 (0.72–1.18)	132 (27.2)	130 (27.2)	116 (24.0)	97 (20.4)	1.08 (0.86–1.36)
Q3	213 (24.6)	104 (23.2)	85 (25.2)	78 (29.0)	1.33 (1.06–1.69)	115 (23.7)	110 (23.0)	131 (27.2)	124 (26.2)	1.42 (1.13–1.78)
Q4	141 (16.3)	139 (31.0)	107 (31.8)	85 (31.6)	1.91 (1.50–2.42)	90 (18.5)	101 (21.1)	118 (24.5)	163 (34.4)	1.94 (1.54–2.44)
*P* for trend					< 0.001					< 0.001
After PS matching										
Each 1-SD increase	673	295	211	167	1.36 (1.22–1.50)	358	364	288	336	1.33 (1.21–1.47)
Quartiles										
Q1	187 (27.8)	69 (23.4)	56 (26.5)	24 (14.4)	1 (Reference)	98 (27.4)	108 (29.7)	77 (26.8)	53 (15.8)	1 (Reference)
Q2	190 (28.2)	71 (24.0)	38 (18.0)	39 (23.4)	0.98 (0.73–1.31)	95 (26.5)	103 (28.3)	66 (22.9)	74 (22.0)	1.12 (0.85–1.48)
Q3	176 (26.2)	63 (21.4)	54 (25.6)	46 (27.5)	1.27 (0.95–1.70)	87 (24.3)	80 (22.0)	85 (29.5)	87 (25.9)	1.49 (1.14–1.96)
Q4	120 (17.8)	92 (31.2)	63 (29.9)	58 (34.7)	2.04 (1.53–2.72)	78 (21.8)	73 (20.0)	60 (20.8)	122 (36.3)	2.01 (1.52–2.65)
*P* for trend					< 0.001					

*PS* propensity score, *8*-*OHdG* 8-hydroxy-2′-deoxyguanosine, *LMCAD* left main coronary artery disease

^a^Models were adjusted for age, sex, smoking habit, alcohol drinking habit, overweight/obesity, hypertension, dyslipidemia., FPG, HbA1c, diabetes duration, and type of diabetes management

^b^The median (interquartile range) of modified Gensini scores before and after PS matching was 27 (20–36) and 27 (20–35.5), respectively

### mtDNA 8-OHdG and cardiometabolic phenotypes

We totally tested 11 biomarkers covering five categories of cardiometabolic phenotypes, i.e. blood lipids (LDL-C, HDL-c, TC, and TG), blood glucose (FPG, HbA1c), blood pressure (systolic and diastolic), proinflammatory measures (CRP and D-dimer), and adiposity measure (BMI). Overall, greater mtDNA 8-OHdG levels was significantly correlated with higher CRP levels (β = 0.182, *P* = 0.005), marginally with lower HDL-c levels (β = − 0.010, *P* = 0.059), but not with other 9 biomarkers after adjustment for confounders (Table 5). In the PS-matched cohort, only the association between mtDNA 8-OHdG and CRP remained significant.

**Table 5:** Associations of mtDNA 8-OHdG with 11 cardiovascular biomarkers in patients with T2DM

Variables	All participants			After PS matching		
	N	β (95% CI)^a^	*P*^a^	N	β (95% CI)^a^	*P*^a^
Blood lipids						
TG (mmol/L)	1920	0.035 (− 0.007, 0.078)	0.100	1346	0.046 (− 0.006, 0.098)	0.081
TC (mmol/L)	1920	0.002 (− 0.044, 0.048)	0.925	1346	− 0.001 (− 0.057, 0.054)	0.962
LDL-c (mmol/L)	1920	0.014 (− 0.023, 0.050)	0.462	1346	− 0.008 (− 0.020, 0.005)	0.251
HDL-c (mmol/L)	1920	− 0.010 (− 0.020, 0.000)	0.059	1346	0.011 (− 0.032, 0.053)	0.498
Blood glucose						
FPG (mmol/L)	1920	0.040 (− 0.038, 0.118)	0.319	1346	0.001 (− 0.085, 0.087)	0.987
HbA1c (%)	1920	0.000 (− 0.053, 0.053)	0.996	1346	− 0.038 (− 0.148, 0.071)	0.492
Blood pressure						
SBP (mmHg)	1920	1.971 (− 0.210, 4.043)	0.077	1346	1.297 (− 0.186, 2.780)	0.086
DBP (mmHg)	1920	0.725 (− 0.124, 1.575)	0.094	1346	0.718 (− 0.277, 1.714)	0.157
Inflammation						
CRP (mg/L)	*1920*	*0.182 (0.055, 0.309)*	*0.005*	*1346*	*0.172 (0.028, 0.315)*	*0.019*
D-dimer (mg/L)	1798	− 0.019 (− 0.067, 0.028)	0.431	1258	0.000 (− 0.057, 0.055)	0.980
Adiposity						
BMI (m/kg^2^)	1920	0.068 (− 0.016, 0.153)	0.111	1346	0.045 (− 0.162, − 0.252)	0.670

Italic values indicate significant results with *P* < 0.05

*BMI* body mass index, *FPG* fasting plasma glucose, *HbA1c* glycosylated haemoglobin, *TC* total cholesterol, *LDL*-*c* low-density lipoprotein cholesterol, *HDL*-*c* high-density lipoprotein cholesterol, *TG* triglycerides, *SBP* systolic blood pressure, *DBP* diastolic blood pressure, *CRP* C-reactive protein

^a^Multivariable linear regression was performed with the mtDNA 8-OHdG as the independent variable and each cardiovascular biomarker as the dependent variable, with adjustment for age, sex, smoking habit, alcohol drinking habit, overweight/obesity, hypertension, dyslipidemia., diabetes duration, and type of diabetes management

### mtDNA 8-OHdG and 1-year clinical outcomes after coronary revascularization

During a median follow-up of 12.4 (IQR: 11.7–12.6) months, 109 (15.5%) MACCEs occurred in 701 diabetic patients who underwent coronary revascularization (PCI: n = 475; CABG: n = 226). After adjustment for confounders, high level of mtDNA 8-OHdG was estimated as an independent predictor for MACCEs (HR = 1.59, 95% CI 1.33–1.90), especially for all-cause death (HR = 2.06) and cardiac death (HR = 1.92, all *P* < 0.001, Table 6). The time-dependent ROC at 12 months suggested that the optimal cut-off value of mtDNA 8-OHdG was 3.22 ∆ct for predicting MACCEs, with an AUC of 0.66, a sensitivity of 0.72, and a specificity of 0.55. Patients with mtDNA 8-OHdG values of > 3.22 ∆ct had a multivariable-adjusted HR of 2.74 (95% CI 1.80–4.17, *P* < 0.001, Table 6) for predicting MACCEs, compared with those without. When stratified by revascularization methods, the HRs for MACCE, all-cause death, and cardiac death were associated with increasing mtDNA 8-OHdG in the PCI group; there were positive associations of mtDNA 8-OHdG with MACCEs and all-cause death in the CABG group (Additional file 3: Table S1).

**Table 6:** Associations of continuous and dichotomous mtDNA 8-OHdG with 1-year outcomes after coronary revascularization

Outcomes	Continuous mtDNA 8-OHdG		Dichotomous mtDNA 8-OHdG			
	HR (95%CI)^a^	*P*^a^	No. of events^b^		HR (95%CI)^a^	*P*^a^
			High (n = 344)	Low (n = 357)		
MACCE	1.59 (1.33–1.90)	< 0.001	78 (22.7)	31 (8.7)	2.74 (1.80–4.17)	< 0.001
All-cause death	2.06 (1.59–2.66)	< 0.001	34 (9.9)	11 (3.1)	3.36 (1.69–6.69)	< 0.001
Cardiac death	1.92 (1.33–2.78)	< 0.001	19 (5.5)	5 (1.4)	3.80 (1.40–10.3)	0.009
Repeat revascularization	1.26 (0.98–1.62)	0.078	47 (13.7)	19 (5.3)	2.67 (1.55–4.57)	< 0.001
Non-fatal MI	1.42 (0.91–2.22)	0.126	12 (3.5)	7 (2.0)	2.30 (0.89–5.92)	0.085
Non-fatal stroke	1.45 (0.59–3.58)	0.417	3 (0.9)	2 (0.6)	1.71 (0.27–10.8)	0.569

*MACCE* major adverse cardiovascular and cerebral events, *MI* myocardial infarction

^a^Models were adjusted for age, sex, smoking habit, alcohol drinking habit, overweight/obesity, hypertension, dyslipidemia., FPG, HbA1c, diabetes duration, and type of diabetes management

^b^The cutoff that categorized the value of mtDNA 8-OHdG as high or low was 3.22 ∆ct based on the results of time-dependent ROC

### In vitro experiments for mtDNA oxidative damage in HUVECs under hyperglycemic conditions

**Fig. 4:**
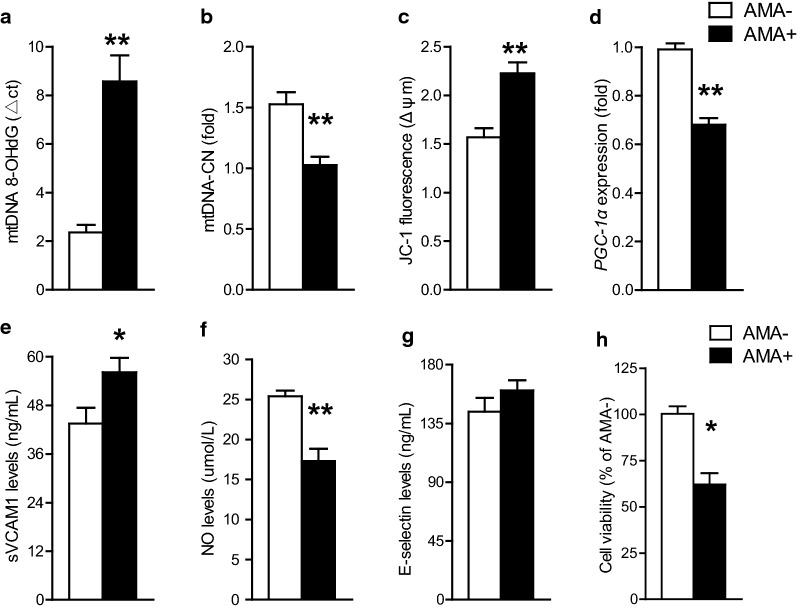
Effects of exposure to antimycin A, an inducer for mtDNA oxidative damage, on mtDNA 8-OHdG (**a**), mtDNA-CN (**b**), mitochondrial membrane potential (**c**), *PGC1*-*α* mRNA expression (**d**), sVCAM-1 levels (**e**), NO levels (**f**), E-selectin levels (**g**), and cell viability (**h**) in glucose-cultured HUVECs. Data are mean ± SD of three independent experiments. *P < 0.05, **P < 0.01 compared with the AMA-untreated cells

To provide in vitro evidence for the nature of the associations reported herein, we tested the effects of AMA, an inducer of mtDNA oxidative damage, in HUVECs under hyperglycemic conditions. As presented in Fig. 4a, exposure to 50 mM of AMA for 24 h led to a 3.7-times increase in mtDNA 8-OHdG compared with that in untreated cells, supporting the direct effect of AMA on mtDNA oxidative damage. Pretreatment of AMA also resulted in adverse alterations in markers of mitochondrial (i.e. decreased mtDNA-CN and *PGC*-*1α* expression, increased ∆ψm magnitude, Fig. 4b–d) and endothelia dysfunction (i.e. decreased NO levels and increased sVCAM-1 levels, Fig. 4e, f). Meanwhile, the cell viability in AMA-treated HUVECs was 37% lower than that in untreated HUVECs (Fig. 4h).

## Discussion

To our knowledge, this is the first study to explore the association of 8-OHdG in leukocyte mtDNA with CAD in diabetic patients. First, in a cross-sectional design covering 1920 patients with T2DM from two provinces in China, mtDNA 8-OHdG was positively associated with CAD presence, coronary stenosis severity, and CRP levels. Then, in 701 prospectively followed diabetic patients, mtDNA 8-OHdG was suggested as a significant predictor of 1-year MACCEs after coronary revascularization. Finally, in vitro experiments showed that exposure to AMA, an inducer of mtDNA oxidative damage, led to adverse alterations in biomarkers of mitochondrial and endothelia dysfunction in HUVECs.

Previous studies consistently showed excreted levels of 8-OHdG in serum, plasma, or urine, mainly reflecting nuclear DNA damage, in association with coronary atherosclerosis in T2DM [43, 44]. This may be partly explained by the generally high caloric intake of diabetic patients with atherosclerotic cardiovascular disease, leading to an increase in mitochondrial energy synthesis, over-production of mitochondrial ROS, and consequently mtDNA oxidative damage in endothelial cells [45, 46]. However, large population studies linking mtDNA oxidative damage to CAD in T2DM are still sparse. A small case–control study involving 275 patients with diabetes or clinical atherosclerosis observed a significant association of mtDNA adducts in blood mononuclear cells with vascular function [47]. In our previous work, mtDNA-CN in leukocytes, while not a direct marker for mtDNA damage, was suggested as a risk factor for CAD, independent of diabetic status [18]. In the present study, we extended our previous work by directly measuring 8-OHdG in mtDNA, which had the advantage of straightly reflecting a steady-state level of oxidative DNA damage occurring within mitochondria. Besides the observation that the linear association between mtDNA 8-OHdG and CAD presence in patients with T2DM remained significant after adjusting for multiple cardiovascular risk factors and T2DM-related variables, we also found that adding mtDNA 8-OHdG into established risk prediction models improved risk discrimination and reclassification for CAD events. Recently, a case-series study of patients who underwent virtual histology intravascular ultrasound also suggested that elevated mtDNA damage in leukocytes was correlated with increased number of thin-capped fbroatheromas, which is a key feature of high-risk plaques [48]. These findings, combined with further in vivo evidence that mtDNA damage occurred prior to plaque formation in *ApoE*-null mice [48], suggest a causative role for mtDNA 8-OHdG as a risk factor for CAD in patients with T2DM.

The previous studies may not be large enough to observe the effect modification by smoking status on mtDNA oxidative damage in T2DM. So the current observation that the association between mtDNA 8-OHdG and CAD presence in diabetic patients was most significant for current smokers, but no longer significant for never smokers was meaningful. After all, a major contribution of cigarette smoking in the pathogenesis of diabetic macrovascular complications is the over-production of ROS, which may aggravate mtDNA oxidative damage and greatly increase CAD risk in patients with T2DM [49]. In population studies, smoking has been associated with increased mtDNA-CN and decreased activity of mitochondrial respiratory chain complexes in a dose-dependent manner [50, 51]. In vitro, the exposure to nicotine-derived nitrosamine ketone, a major toxic ingredient of cigarette smoke, may lead to imbalanced cellular redox status, defective mitochondrial biogenesis, and impaired energy production in mitochondria [52]. Taken together, our data support the view that cigarette smoking may modify the effects of mtDNA oxidative damage on the development of CAD in patients with T2DM.

A novel finding of our study is that mtDNA 8-OHdG was a significant predictor for composite MACCEs, especially for all-cause mortality and cardiac mortality after revascularization in patients with T2DM. This result was supported by our further observation that mtDNA 8-OHdG positively correlated with coronary stenosis severity, as determined by the number of diseased vessels and modified Gensini scores. Indeed, functional studies have suggested that mtDNA oxidative damage under diabetic conditions may trigger a series of pathological events including abnormal mitochondrial gene transcription, uncontrolled oxidative stress, and endothelial dysfunction [12, 53], all of which contribute to plaque progression and rupture [54]. In vitro, hyperglycemia may also induce the interaction of mtDNA oxidative damage with inflammatory cytokine release, causing apoptosis of monocytes, T2DM-related endothelial dysfunction, and plaque instability [48]. In line with this evidence, we found that mtDNA 8-OHdG positively correlated with CRP levels, a well established measure of systemic inflammation, in patients with T2DM, and that AMA-induced mtDNA oxidative damage was linked to adverse alterations in markers of endothelial dysfunction and cell viability in high glucose-cultured HUVECs. Together, these findings suggest that mtDNA oxidative damage is on a chain from cardiovascular morbidity to mortality in patients with T2DM.

The pivotal effect of elevated mtDNA 8-OHdG on obstructive CAD in T2DM raises the question of whether this effect is mediated by possible lifestyle or genetic factors. Recent evidence from aged mice showed that increased mitochondrial oxidative stress might induce age-associated vascular wall remodeling, causing aortic stiffening and cardiovascular disease [55, 56], whereas supplementation with cobalamin (i.e. vitamin B12) and folate could improve mitochondrial oxidative capacity to protect the failing heart in mice [57]. There was also ex vivo evidence showing that a dominant negative *Ogg1* mutant F460A restored Ogg1 activity, and, consequently reduced mtDNA damage under the hyperglycemic milieus [15]. So, human studies, specifically exploring the mediation of mtDNA 8-OHdG variations by aging, B-vitamin nutrition status, and *OGG1* mutations, would be of interest.

Our study has limitations. First, although we used a series of rigorous statistical approaches to minimize the effect of residual confounding, we could not establish a causal relationship between mtDNA 8-OHdG and obstructive CAD owing to our observational design. Second, our study only enrolled high-risk participants who were referred for coronary angiography. This may cause the selection bias that influences the stability of results. Third, further assessment of the dose–response effect of smoking and mtDNA 8-OHdG was not available, due to lack of collection of more detailed information on cigarette smoking such as pack-years of smoking or smoking duration. Fourth, the observation of the significant association between mtDNA 8-OHdG and CRP levels should be interpreted cautiously, because all cardiovascular biomarkers were measured at a single time point, which might bias the results. Finally, 1 year of follow-up was not long enough to assess the association of mtDNA 8-OHdG with long-term outcomes after coronary revascularization.

## Conclusions

In summary, our data show that higher mtDNA 8-OHdG was cross-sectionally associated with increased odds of obstructive CAD, higher degree of coronary stenosis, and higher CRP levels, and prospectively with 1-year adverse clinical outcomes after coronary revascularization. Future prospective studies in seeking mtDNA-related biomarkers for incident CAD should focus on direct detection of mtDNA 8-OHdG rather than on other indirect measurements. Our in vitro experiments also suggest that exposure to AMA, an inducer for mtDNA oxidative damage, may correlate with adverse alterations in endothelia function in HUVECs. These results may serve as a basis for future physiological studies to explore whether AMA-induced endothelia dysfunction is directly caused by mtDNA 8-OHdG or mediated by other casual factors.

## Supplementary information

**Additional file 1: Methods.** Assessment of artery stenosis severity and definitions of composites of MACCE.

**Additional file 2: Fig. S1.** Correlations of the data of mtDNA 8-OHdG quantified by aPCR and ELISA in 200 randomly selected participants. R coefficient and P value were obtained from the Pearson correlation test.

**Additional file 3: Table S1.** Associations of mtDNA 8-OHdG with 1-year clinical outcomes stratified by revascularization modality.

**Additional file 4: Data S1.** Original data of all 1920 participants analyzed in this study.

## Data Availability

Data generated or analyzed during this study are included in the Additional file 4: Data S1.

## Abbreviations

8-OHdG: 8-Hydroxy-2′-deoxyguanosine
CAD: Coronary artery disease
T2DM: Type 2 diabetes mellitus
mtDNA: Mitochondrial DNA
ROS: Reactive oxygen species
BMI: Body mass index
FPG: Fasting plasma glucose
HbA1c: Glycosylated haemoglobin
CRP: C-reactive protein
PS: Propensity score
MACCE: Major adverse cardiovascular and cerebrovascular events
HUVECs: Human umbilical vein endothelial cells
