# Supplementary material for: Mitochondrial 8-hydroxy-2′-deoxyguanosine and coronary artery disease in patients with type 2 diabetes mellitus
Source: Cardiovasc Diabetol. 2020 Feb 19;19:22. doi: 10.1186/s12933-020-00998-6 (PMC7029479; doi:10.1186/s12933-020-00998-6)
Supplement: Supplementary file 1 — Additional file 1: Methods. Assessment of artery stenosis severity and definitions of composites of MACCE. [file 12933_2020_998_MOESM1_ESM.pdf]

Definitions of composites of MACCE

All-cause death was defined as death from any cause. Cardiac death was defined as any death due to immediate cardiac cause, procedure-related deaths, and deaths without a clear noncardiac cause. MI was defined as ischaemic signs or symptoms and new pathological Q-waves in ≥2 contiguous ECG leads, or/and an elevation CK-MB or troponin above the 99th percentile limit of normal and at least ≥20% above the most recent value. Stroke was defined as a focal neurologic deficit of central origin lasting >72 hours, or a focal neurologic deficit of central origin lasting >24 hours, with imaging evidence of cerebral infarction or intracerebral hemorrhage. Repeat revascularization was defined as any repeat PCI or CABG. All stages of a staged index PCI procedure would be considered part of the index revascularization procedure and not a repeated revascularization.
