# Supplementary material for: Mitochondrial 8-hydroxy-2′-deoxyguanosine and coronary artery disease in patients with type 2 diabetes mellitus
Source: Cardiovasc Diabetol. 2020 Feb 19;19:22. doi: 10.1186/s12933-020-00998-6 (PMC7029479; doi:10.1186/s12933-020-00998-6)
Supplement: Supplementary file 3 — Additional file 3: Table S1. Associations of mtDNA 8-OHdG with 1-year clinical outcomes stratified by revascularization modality. [file 12933_2020_998_MOESM3_ESM.pdf]

Table S1. Associations of mtDNA 8-OHdG with 1-year clinical outcomes stratified by revascularization modality.

	PCI group (n = 475)		CABG group (n = 226)		
**Clinical outcome**	**HR (95%CI)** [a]	***P*** [a]	**HR (95%CI)** [a]	***P*** [a]	$P_{interaction}$ [b]
MACE	1.57 (1.28-1.93)	< 0.001	1.58 (1.12-2.22)	0.009	0.98
All-cause death	2.00 (1.51-2.67)	< 0.001	1.73 (1.00-2.98)	0.048	0.64
Cardiac death	2.01 (1.33-3.03)	< 0.001	1.33 (0.63-2.80)	0.454	0.34
Repeat revascularization	1.28 (0.94-1.73)	0.114	1.48 (0.89-2.46)	0.131	0.63
MI/stroke	1.35 (0.89-2.05)	0.159	1.64 (0.43-6.33)	0.472-	0.79

[a] Models were adjusted for age, sex, smoking habit, alcohol drinking habit, overweight/obesity, hypertension, dyslipidemia., FPG, HbA1c, diabetes duration, and type of diabetes management.

[b] $P_{interaction}$ was obtained from the $\chi 2$-based Q test.
